# Impact of Glycosidic Bond Configuration on Short Chain Fatty Acid Production from Model Fermentable Carbohydrates by the Human Gut Microbiota

**DOI:** 10.3390/nu9010026

**Published:** 2017-01-01

**Authors:** Hannah C. Harris, Christine A. Edwards, Douglas J. Morrison

**Affiliations:** 1 School of Medicine, Dentistry and Nursing, College of Medical Veterinary and Life Sciences, University of Glasgow, Glasgow G31 2ER, UK; hannahcharris1@gmail.com (H.C.H.); Christine.Edwards@glasgow.ac.uk (C.A.E.); 2 Scottish Universities Environmental Research Centre, University of Glasgow, Glasgow G75 0QF, UK

**Keywords:** colonic fermentation, gut microbiota, non-digestible carbohydrates, short chain fatty acids, glycosidic bond

## Abstract

Short chain fatty acids (SCFA) are the major products of carbohydrate fermentation by gut bacteria. Different carbohydrates are associated with characteristic SCFA profiles although the mechanisms are unclear. The individual SCFA profile may determine any resultant health benefits. Understanding determinants of individual SCFA production would enable substrate choice to be tailored for colonic SCFA manipulation. To test the hypothesis that the orientation and position of the glycosidic bond is a determinant of SCFA production profile, a miniaturized in vitro human colonic batch fermentation model was used to study a range of isomeric glucose disaccharides. Diglucose α(1-1) fermentation led to significantly higher butyrate production (*p* < 0.01) and a lower proportion of acetate (*p* < 0.01) compared with other α bonded diglucoses. Diglucose β(1-4) also led to significantly higher butyrate production (*p* < 0.05) and significantly increased the proportions of propionate and butyrate compared with diglucose α(1-4) (*p* < 0.05). There was no significant effect of glycosidic bond configuration on absolute propionate production. Despite some differences in the SCFA production of different glucose disaccharides, there was no clear relationship between SCFA production and bond configuration, suggesting that other factors may be responsible for promoting selective SCFA production by the gut microbiota from different carbohydrates.

## 1. Introduction

There is increasing evidence that bioactive molecules produced during colonic bacterial fermentation play a central role in the beneficial effects of non-digestible carbohydrates (NDC) [1]. Dietary NDC are the major fermentable component of dietary fiber and have been associated with positive health outcomes related to appetite regulation, body composition and metabolic health [2,3]. Short chain fatty acids (SCFA) are the main products of saccharolytic bacterial fermentation of NDC, with acetate, propionate, and butyrate being the principal SCFA produced [4]. Although there is considerable variability between individuals, SCFA are produced in the approximate ratio 60:20:20 for acetate, propionate and butyrate respectively [5]. With increased interest in the effects of SCFA on human metabolic health [2], there is a growing need to understand how NDCs and the gut microbiota interact to yield different patterns of SCFA production and if certain profiles are related to improved health outcomes.

Acetate is a central metabolite in intermediary metabolism and is involved in lipid and cholesterol synthesis [6,7] and central appetite regulation [8]. In addition to inhibiting de novo cholesterol and lipid synthesis [9,10], propionate is a precursor for intestinal [3] and hepatic gluconeogenesis [7]. Selectively increasing colonic propionate in humans, through the consumption of inulin propionate ester, has been shown to regulate appetite, reduce hepatic and intra-abdominal visceral fat depots and reduce body weight gain in overweight adults [2]. Butyrate is the preferred energy source for the colonic epithelial cells [11], has regulatory roles in cellular proliferation and apoptosis [12] and has recently been shown to play a role in immune regulation [13,14]. At the molecular level, some of the observed effects of SCFA on cellular function are thought to be mediated through a receptor mediated cascade involving free fatty acid receptor 2 (FFAR2) and free fatty acid receptor 3 (FFAR3) [15]. These receptors are present on a range of cell types including endocrine cells, adipocytes and immune cells [16]. FFAR 2/3 expressed on enteroendocrine L-cells have been shown to be involved in the receptor mediated release of the anorexigenic gut hormones peptide YY (PYY) and glucagon-like peptide 1 (GLP-1) [17]. On immune cells FFAR2 appears to play an important role in the normal resolution of inflammatory responses in animal models of colitis, arthritis and asthma [18].

The myriad of metabolically important and immune-related cell types expressing FFAR 2/3 has led to a renewed interest in the role of SCFA as important signaling molecules linking diet, the gut microbiome and health [19]. However, at present, it is not straightforward to selectively manipulate production of individual SCFA in the diet of humans because the factors which drive the production of one SCFA over another are not well understood.

The profile of SCFA production is thought to be related to the physicochemical properties of NDCs reaching the colon, which in turn may determine which members of the gut microbiota consortium are able to derive energy from NDC fermentation, yielding SCFA as the reduced end-products [20]. Factors which have been shown to influence SCFA production profile include monosaccharide composition and distribution, glycosidic bond linkage and NDC chain length and branching [21,22,23]. Soluble NDC appear more readily fermented than insoluble NDC, and previous studies have suggested that solubility (or chain length) may influence the profile of SCFA produced [24]. Pyrodextrinised starch, a low molecular weight carbohydrate with new β-glycosidic bonds formed after heat treatment of the native starch, has been shown to increase propionate production in vitro [24]. Furthermore, in vitro investigations have also demonstrated a linear association between the amount of soluble fiber and SCFA production [25]. However, the relationship between NDC solubility and SCFA production is not truly representative of the human diet and its mixed NDC composition, exemplified by the fact that insoluble, high molecular weight resistant starch is abundant in the human diet and highly fermentable by the human gut microbiota [26].

Fermentation of some mono- and di-saccharides appears to favor selective SCFA production. In vitro, sorbitol fermentation selectively increased butyrate production whereas glucose, xylose, and fructose fermentation selectively increased acetate production [21,23]. Rhamnose has been shown, in both in vitro fermentations and in vivo feeding studies, to selectively increase propionate production [27,28]. Lactose fermentation selectively increased acetate production [23,29]. In an experiment with a single fecal donor, it was observed that diglucose β(1-2) (sophorose), and mannobiose with different glycosidic bond linkages (dimannose 2α, 3α, 4α, 6α) increased propionate production. Diglucose α(1-1) α(α, α-trehalose) and diglucose β(1-1) β(β, β-trehalose) and 3α-digalactose (3α-galactobiose) increased butyrate production [29].

Predicting SCFA yield from individual NDC is not straightforward. Acetate is almost exclusively the most abundant SCFA produced. Propionate production is associated with greater abundance of β-glycosidic bonds in NDCs as demonstrated by fermentation of laminarin [30] and pyrodextrinised starch [24]. In contrast, starch consisting of α-glycosidic bonds, and oligofructose consisting of both α and β-glycosidic bonds results in a selective increase in butyrate production [24,25]. SCFA production is also influenced by the composition of these complex NDC where the fermentability of each of the constituent sugars may determine the differences in SCFA produced [22]. Colonic bacteria differ in their ability to utilize substrates, yielding often specific SCFAs through the saccharolytic fermentation pathways encoded in their genomes [31]. For example, the genus *Roseburia* utilizes starch to produce butyrate [32] and in contrast both, *Ruminococcus obeum* related species and *Roseburia inulinivorans* are involved in the production of propionate [33]. The relationship between monosaccharide composition, intra-molecular glycosidic bond configuration and chain length appears to be complex. However, attempts to model the fermentation process in silico have shown some promise. Using a differential equation model, many of the changes in the microbiota and key metabolites, such as SCFA, have been simulated in continuous flow fermenters inoculated with human fecal microbiota [34].

In the present study, the relationship between glycosidic bond orientation and position and SCFA production was investigated using glucose-glucose disaccharides as model NDC substrates. This allowed for experimental control of confounding factors such as solubility and monosaccharide compositions on SCFA production.

## 2. Materials and Methods

### 2.1. Substrates

Glucose-glucose disaccharides (diglucoses) were used as model substrates to explore all bond options on SCFA production with the exception of diglucose β(1-1) β (β, β-trahalose) which was not commercially feasible. Disaccharide substrates were obtained from Carbosynth (Berkshire, UK). Bond linkages investigated were; diglucose α(1-1) (α, α-d-trehalose dihydrate), diglucose β(1-1) (α, β-trehalose), diglucose α(1-2) (kojibiose), diglucose β(1-2) (sophorose), diglucose α(1-3) (nigerose), diglucose β(1-3) (laminaribiose), diglucose α(1-4) (d-maltose monohydrate), diglucose β(1-4) (d-cellobiose), diglucose α(1-6) (isomaltose), diglucose β(1-6) (d-gentiobiose). A blank (no-substrate) control was also used to adjust for background SCFA production. Due to the cost of some substrates, miniaturized batch fermentations of those used previously were established [35,36]. In the present study, the method was proportionally scaled down by a factor of 20 (to 2.5 mL) and validated against our standard larger (50 mL) fermentation system.

### 2.2. Batch Fermentations

Fermentation vials contained: 50 mg of each substrate, 0.1 mL of reducing solution (per 50 mL 312.5 mg cysteine hydrochloride, 2 mL 1 M NaOH, 312.5 mg Na_2_S·9H_2_O, dH_2_O to 50 mL) and 2.1 mL of pre-boiled, and cooled under oxygen free nitrogen (OFN) fermentation medium (per liter 2.25 g tryptone, 450 mL dH_2_O, 112.5 μL micromineral solution (13.2 g CaCl_2_·2H_2_O, 10 g MnCl_2_·4H_2_O, 1 g CoCl_2_·6H_2_O, 8 g FeCl_3_·6H_2_O to 100 mL using dH_2_O), 225 mL bicarbonate solution (2 g NH_4_HCO_3_, 17.5 g NaHCO_3_ to 500 mL with dH_2_O), 225 mL macromineral solution (providing buffering) (2.85 g Na_2_HPO_4_, 3.1 g KH_2_PO_4_, 0.3 g MgSO_4_·7H_2_O to 500 mL with dH_2_O) and 1.125 mL 0.1% resazurin (an indicator of anaerosis), and adjusted for a starting fermentation pH of 7 was sealed with airtight seals and degassed under oxygen free nitrogen (OFN) for one minute. Per vessel, 250 μL of 32% fecal slurry prepared by adding pre-boiled and OFN cooled sodium phosphate Sorensen’s buffer to a homogenized stool and blended (with a standard household blender) and strained through nylon (to remove large food residues) before being injected into the vial. The vials underwent a further degassing with OFN for 1 min before being incubated in a shaking water bath at 37 °C. After 0, 8 and 24 h of incubation, 400 μL of fermentation fluid were taken for pH measurement (Mettler Toledo pH meter), and 150 μL of 1 M NaOH added to stabilize the SCFA before extraction.

### 2.3. Participants

Stool samples were obtained from 15 healthy Caucasian individuals (8 females, 7 males, median age, 28; 20–52 years), who had not taken antibiotics in the previous 6 months and had no gastrointestinal disease. Ethical permission was granted by the College of Medical, Veterinary and Life Sciences Ethics Committee, University of Glasgow (Application No.: 2011023) with the sample providers giving informed signed consent. Stool samples were prepared and incubated within 2 h of being voided.

### 2.4. SCFA Extraction and Analysis

For SCFA extraction, 200 μL of the fermentation fluid were extracted. Orthophosphoric acid (50 μL) and 50 μL internal standard (2-ethyl butyric acid in 2 M NaOH; 73.78 mM) were added and mixed by vortexing. To this mixture, 1 mL of diethyl-ether was added and vortexed for 1 min. The ether phase was removed, and the ether extraction repeated twice more with the three extracts combined and stored in gas-tight vials at −20 °C until analysis by gas chromatography with flame ionization detection (GC-FID). SCFA concentrations were calculated using the ratio of each SCFA to the internal standard (73.8 mM, 2-ethylbutyric acid), calibrated for individual SCFA response in the detector using an external standard acetic acid (183.51 mM), propionic acid (132.52 mM), butyric acid (107.06 mM), valeric acid (88.63 mM), caproic acid (74.36 mM), enanthic acid (66.25 mM), caprylic acid (57.84 mM), isobutyric acid (104.13 mM), isovaleric acid (86.59 mM) and isocaproic acid (50.93 mM), all in 2 M NaOH.

Statistical analysis was conducted using SPSS version 22 (IBM, Chicago, IL, USA). The Shapiro Wilk test was used to test for normality of the data. One-way ANOVA with post hoc Bonferroni analysis were carried out on log transformed data on all occasions. Statistical significance was identified at *p* < 0.05. Statistical power was measured by post hoc power analysis using Minitab Inc. Version 16 (Minitab Inc., State College, PA, USA). With 10 incubations the study was powered (0.8) to observe a 9.5 mM (0.47 mmol/g carbohydrate/day) difference in propionate production, an additional 5 incubations provided the power (0.8) to observe a 7.5 mM (0.37 mmol/g carbohydrate/day) difference in propionate production. Previous in vitro fermentation studies have shown similar differences in propionate production between different fibers [35] and between simple sugars [21].

## 3. Results

### 3.1. Effects of Glycosidic Bond Position on Fermenter pH

For all substrates tested the initial pH of the fermentation system did not differ significantly (Table 1). At both 8 and 24 h of fermentation, the pH for diglucose α(1-1) was significantly higher than all other substrates in the α orientation (*p* < 0.05) and diglucose β(1-1), (*p* < 0.01). In the β orientation, β(1-6) had the lowest pH at 24 h which was significantly lower than both β(1-3) and β(1-4) (*p* < 0.05).

### 3.2. Effects of Glycosidic Bond Position on SCFA Production

**Figure 1:**
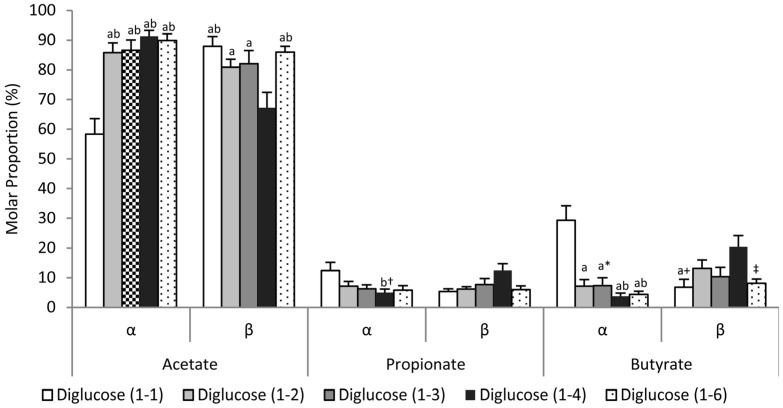
Molar proportions of short chain fatty acids (SCFA) production after 24 h of fermentation. Mean (SEM), α(1-1), β(1-4) *n* = 15, β(1-1) *n* = 9 all others *n* = 10. ^a^ indicates significant differences to α(1-1), ^b^ indicates significant differences from β(1-4) *p* < 0.05. ^†^ indicates *p* = 0.086 vs. α(1-1) * indicates *p* = 0.057 vs. β(1-4), ^+^ indicates *p* = 0.051 vs. β(1-4), ^‡^ indicates *p* = 0.078 vs. α(1-1).

There was no difference in total SCFA production as a result of different glycosidic bond position, however varying glycosidic bond position led to significant differences in acetate and butyrate production (Table 2). When considering glycosidic bonds in the α orientation, diglucose α(1-1) produced significantly more (mean (SEM)) butyrate compared with diglucose α(1-3) (0.9 (0.2) vs. 0.2 (0.1) mmol/g carbohydrate/day, *p* = 0.024), diglucose α(1-4) (0.9 (0.2) vs. 0.1 (0.0) mmol/g carbohydrate/day, *p* = 0.001) and diglucose α(1-6) (0.9 (0.2) vs. 0.2 (0.0) mmol/g carbohydrate/day, *p* = 0.006) and significantly less acetate compared with diglucose α(1-4) (1.8 (0.3) vs. 2.9 (0.3) mmol/g carbohydrate/day, *p* = 0.009) and diglucose α(1-6) (1.8 (0.3) vs. 2.9 (0.2) mmol/g carbohydrate/day, *p* = 0.011) respectively. Proportionally, diglucose α(1-1) also led to significantly increased butyrate production (*p* < 0.01) and significantly reduced acetate production (*p* < 0.01) compared with all other substrates with α bond configuration (Figure 1).

When considering glycosidic bonds in the β orientation, diglucose β(1-6) led to significantly higher acetate production compared with diglucose β(1-4) (3.7 (0.3) vs. 1.9 (0.2) mmol/g carbohydrate/day, *p* = 0.001) (Table 2). Proportionally, diglucose β(1-4) resulted in significantly reduced acetate production compared with diglucose β(1-1) (67.2 (5.2)% vs. 88.0 (3.3)%; *p* = 0.016) and diglucose β(1-6) (67.2 (5.2)% vs. 86.0 (2.0)%, *p* = 0.023). There was a trend for a higher proportion of butyrate production from diglucose β(1-4) compared with diglucose β(1-1) (20.4 (3.8)% vs. 6.7 (27)%, *p* = 0.051) (Figure 1).

### 3.3. Effects of Glycosidic Bond Orientation (α or β) on SCFA Production

**Figure 2:**
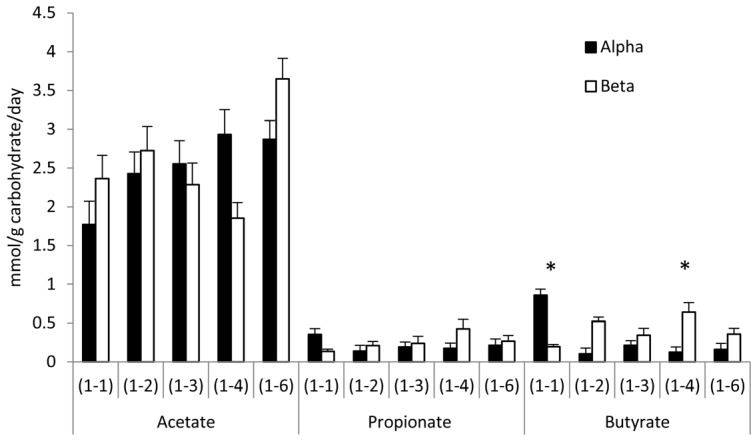
SCFA production after 24 h of fermentation mmol/g carbohydrate/day. Mean (SEM), α(1-1), β(1-4) *n* = 15, β(1-1) *n* = 9 all others *n* = 10. * Significant differences between bond anomers.

The effect of anomeric orientation on SCFA production is illustrated in Figure 2 and Table 2. There were no differences in total SCFA production but diglucoses with α(1-1) bonding increased butyrate production compared with diglucose β(1-1) (0.9 (0.2) vs. 0.2 (0.1) mmol/g carbohydrate/day, *p* = 0.01) whereas diglucose α(1-4) produced less butyrate than diglucose β(1-4) (0.1 (0.0) vs. 0.6 (0.1) mmol/g carbohydrate/day, *p* = 0.038). Proportionally, diglucose β(1-4) also resulted in a significant increased propionate (12.4 (2.8)% vs. 4.9 (1.2)% , *p* = 0.037) and butyrate (20.4 (3.8)% vs. 3.7 (1.1)% , *p* = 0.001), and decreased acetate (67.2 (5.2)% vs. 91.3 (1.9)% , *p* = 0.001) production compared with diglucose α(1-4). Diglucose α(1-1) led to significantly more butyrate (29.3 (4.9) vs. 6.7 (2.7)%, *p* = 0.001) and significantly less acetate production (58.3 (5.2) vs. 88.0 (3.3)%, *p* < 0.001) compared with diglucose β(1-1) (Figure 1).

### 3.4. Subject SCFA Variability

**Figure 3:**
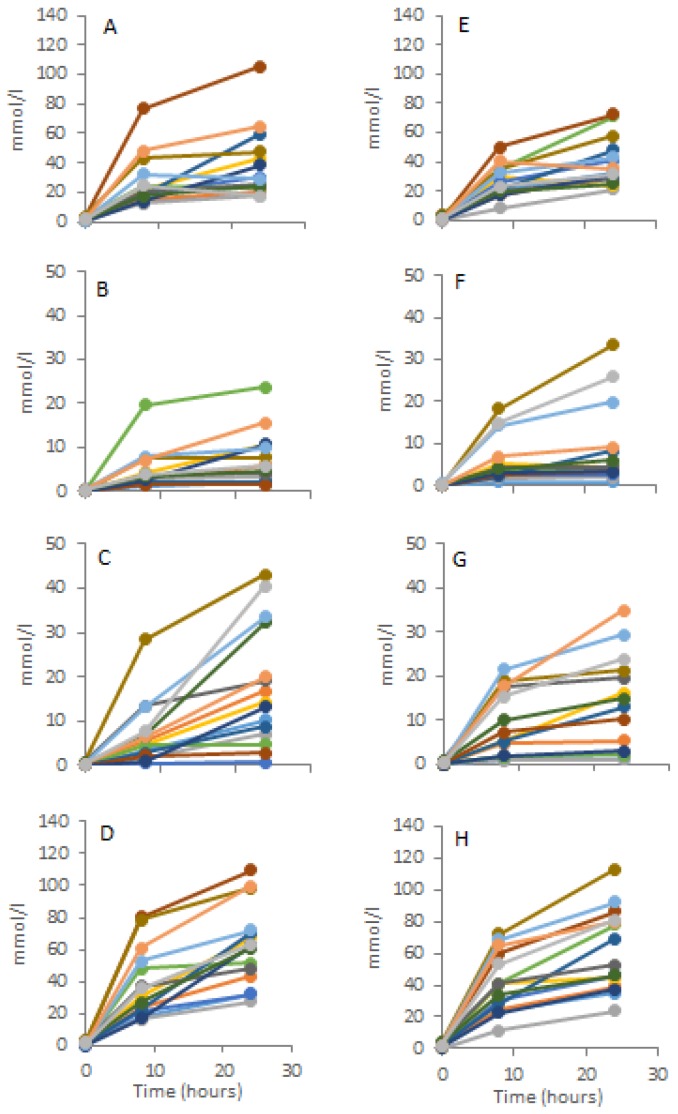
Individually plotted absolute SCFA production. Di-glucose α(1-1): (**A**) Acetate; (**B**) Propionate; (**C**) Butyrate; (**D**) Total SCFA.; Di-glucose β(1-4); (**E**) Acetate; (**F**) Propionate; (**G**) Butyrate; (**H**) Total SCFA.

Significant variability in SCFA production was observed between the individual donor samples. A small subset of donors (*n* = 3) appeared to have much higher propionate production compared with other donors (Figure 3). Butyrate production was also variable between individuals with a small subset of donors (*n* = 4) appearing to have much higher butyrate production. There was no consistent pattern across the substrates and participants for high SCFA yield.

## 4. Discussion

Determining which primary factor(s) drive production of individual SCFA could enable selective manipulation of SCFA production in the colon. Such targeted activation through dietary means would enable selective impact on metabolism and health. For example, Chambers et al. demonstrated that selective increases in propionate production led to improvements in appetite regulation, hepatic and intra-abdominal visceral fat accretion and body weight gain in overweight adults [2]. Although limited amounts of dietary glucose disaccharides are likely to reach the colon due to digestion and/or absorption in vivo, they represent useful model compounds for in vitro studies because they control for other potential confounding factors which can influence SCFA production such as monomer composition and solubility. In the present study, glycosidic bond position had little impact on SCFA production except for diglucose (1-1) which had marked effects on acetate and butyrate production with α(1-1) producing increased butyrate compared to the other α anomers (*p* < 0.05, and for α(1-2); *p* < 0.06), and the lowest acetate (*p* < 0.05 for α(1-1) and α(1-6)). The anomeric orientation on diglucose (1-4) also had modest effects on the production of all three SCFA. Interestingly, there were no significant effects of bond configuration on propionate production. Many of the changes in butyrate production were associated with a reduction in acetate. This is in line with previous isotope based observations that a significant proportion of butyrate production is derived from acetate through inter-conversion [37,38].

As a result of the high cost of the glucose disaccharides used, fermentation experiments were miniaturized and the number of fermentations possible was restricted. Miniaturization is likely have led to increased variability due to propagation of errors when dealing with smaller samples. This may have increased the variability of the data but the study was powered to observe a difference of 9.5 mM (equivalent to 0.47 mmol/g carbohydrate/day) in propionate production from NDC in vitro which also demonstrated an effect on energy intake in humans [39]. The yield of SCFA was lower than expected from stoichiometric equations which may suggest incomplete fermentation of the substrates with the experimental timeframe. This could be supported indirectly by the observed rapid production of SCFA and drop in pH which would become increasingly inhibitory to ongoing fermentation. A subpopulation of individuals did appear to have greater capacity for propionate and butyrate production compared with other sample donors. Previous studies have indicated that SCFA production varies with pH, with lower pH favoring butyrate production and higher pH favoring propionate [32]. In our study, pH was measured but no variations were observed between participants that would explain the higher propionate and butyrate production seen in this small subset of participants. The reasons for this are unclear but variations in bacterial groups involved in propionate and butyrate production have been observed previously [33]. Recent work has also demonstrated that the microbiota responds quickly to dietary changes [40] and the changes in activity observed within the subgroups in this study may reflect variations in dietary intake (which was not controlled) in the days prior to sample collection.

The physicochemical properties of NDC are thought to play a role in determining SCFA production since some NDC appear to selectively increase production of individual SCFA. Changing the physicochemical properties of an NDC can also affect SCFA production. For example, increased solubility as a result of pyrodextrinization of starch, which introduces β bonding into the mainly α bonded starch, led to increased production of propionate in vitro [24]. Other β-glucans have also been associated with increased propionate production including, laminarin (mainly β(1-3) and β(1-6) bonding) and psyllium (mainly β(1-4) bonding) [30]. Resistant starch consisting of α(1-4) and α(1-6) bonding and oligofructose with β(1-2) and α(1-2) bonding are associated with increased butyrate production [36,41,42]. Stewart et al. [43] elegantly demonstrated in the fermentation of inulin type fructans (ITF) that both rate and proportions of SCFAs produced are influenced by chain length. Increased molar ratios of acetate and a decrease in molar ratios of butyrate were observed as ITF chain length increased. No effect was observed for molar proportion of propionate with ITF chain length. Thus whether the selected disaccharides serve as a model for fermentation of larger and more complex oligo and polysaccharides, where both endo- and exo- glycoside hyrolases may play complementary roles in saccharolytic degradation of polymers [44], is not clear. However, the model substrates studied, uniquely allowed the effect of bond position and configuration to be studied free of the confounding effects such as additional bonding configurations invariably present in many oligo- and polysaccharides, solubility and chain length—all potential contributors to changes in the rate and molar proportion of SCFA production.

From the data in the present study, it was clear that the position and configuration of glycosidic bonding in diglucose had no major impact on SCFA production profiles. There was some modest impact on SCFA as a result of anomeric orientation for (1-1) and (1-4) diglucose linkages, with both having ~80% difference in butyrate production between anomers. These data are supported, in part, by previous small-scale studies. Sanz et al. demonstrated, in a single fecal donor utilizing all glucose disaccharides, no trends were associated with diglucose linkages, anomeric formation and the resulting bacterial population. Although the fermentation of (1-1) did not lead to differences in bacterial populations compared to the control, (1-4) fermentation led to a significant increase in bifidobacteria compared with the control [29]. They did however observe some changes in SCFA production. For example, diglucose isomers containing β bonds (including β(1-1)β trehalose which we were unable to include within our investigation) significantly increased propionate and butyrate production compared to the α bonded diglucose isomers. However diglucose β(1-6) bonding did not follow this pattern [29]. Diglucoses α(1-1) also resulted in significantly reduced propionate production, but significantly increased butyrate production compared to other α bonded diglucoses. The reasons for this selective increase in butyrate production with α(1-1) glycosidic bond configuration are not clear and warrant further investigation. In another study, diglucose α(1-4) and diglucose α(1-6) linkages led to non-significant increases in propionate and butyrate production [21]. However, comparisons between these studies and the present study are difficult to interpret because of their short fermentation duration and the low number of subjects.

## 5. Conclusions

In conclusion, our data show that the orientation of glycosidic linkage, between glucose monomers at least, is not the primary factor determining the SCFA production profile. This suggests that the drivers of SCFA production may be related to other facets of carbohydrate structure or microbiology. Further investigations into how other aspects of carbohydrate structure impact upon SCFA production by the microbiota are required.

## Figures and Tables

**Table 1:** pH of the fermenter system after 0, 8 and 24 h of fermentation.

	0 h		8 h		24 h	
	α	β	α	β	α	β
Diglucose (1-1)	7.13 (0.14)	7.08 (0.24)	5.10 (0.17) ^a,^*	4.32 (0.25)	5.51 (0.06) ^a,^*	3.91 (0.12)
Diglucose (1-2)	7.04 (0.21)	7.23 (0.18)	4.43 (0.27) ^b^	4.29 (0.10)	3.93 (0.10) ^b^	3.85 (0.08)
Diglucose (1-3)	7.04 (0.20)	6.98 (0.22)	4.18 (0.18) ^b^	4.50 (0.11)	3.92 (0.10) ^b^	4.05 (0.12) ^b^
Diglucose (1-4)	7.06 (0.21)	7.16 (0.17)	3.93 (0.09) ^b,^*	4.49 (0.09)	3.64 (0.06) ^b,^*	4.15 (0.14) ^b^
Diglucose (1-6)	7.08 (0.21)	7.19 (0.08)	4.11 (0.25) ^b^	4.24 (0.23)	3.81 (0.06) ^b^	3.77 (0.11) ^a^

Mean (SEM), different letters within a column indicate significant differences *p* < 0.05. * indicates differences between bond anomers *p* < 0.01. α(1-1), β(1-4) *n* = 15, β(1-1) *n* = 9 all others *n* = 10.

**Table 2:** SCFA production (mmol/g carbohydrate/day) after 24 h of fermentation.

	Acetate		Propionate		Butyrate		Total	
Glucose Bond Orientation	α	β	α	β	α	β	α	β
Diglucose (1-1)	1.8 (0.3) ^a^	2.4 (0.3)	0.4 (0.1)	0.1 (0.0)	0.9 (0.2) ^a,^*	0.2 (0.1)	3.0 (0.4)	2.7 (0.3)
Diglucose (1-2)	2.4 (0.3)	2.7 (0.3)	0.1 (0.1)	0.2 (0.1)	0.1 (0.1)	0.5 (0.1)	2.9 (0.3)	3.5 (0.5)
Diglucose (1-3)	2.6 (0.3)	2.3 (0.3)	0.2 (0.1)	0.2 (0.1)	0.2 (0.1) ^b^	0.3 (0.1)	3.0 (0.3)	2.9 (0.4)
Diglucose (1-4)	2.9 (0.3) ^b^	1.9 (0.2) ^a^	0.2 (0.1)	0.4 (0.1)	0.1 (0.0) ^b,^*	0.6 (0.1)	3.2 (0.4)	2.9 (0.3)
Diglucose (1-6)	2.9 (0.2) ^b^	3.7 (0.3) ^b^	0.2 (0.1)	0.3 (0.1)	0.2 (0.0) ^b^	0.4 (0.1)	3.2 (0.3)	4.3 (0.3)

Mean (SEM), different letters within a column indicate significant differences *p* < 0.05. * indicates differences between bond anomers *p* < 0.05, α(1-1), β(1-4) *n* = 15, β(1-1) *n* = 9 all others *n* = 10.

## Abbreviations

The following abbreviations are used in this manuscript:

FFAR2/3	Free fatty acid receptor 2/3
GC-FID	Gas chromatography flame ionization detector
GLP-1	glucagon-like peptide-1
NDC	non-digestible carbohydrate
OFN	oxygen free nitrogen
PYY	peptide YY

## References

[1] Den Besten G., van Eunen K., Groen A.K., Venema K., Reijngoud D.-J., Bakker B.M. (2013). The role of short-chain fatty acids in the interplay between diet, gut microbiota, and host energy metabolism. J. Lipid Res..

[2] Chambers E.S., Viardot A., Psichas A., Morrison D.J., Murphy K.G., Zac-Varghese S.E.K., MacDougall K., Preston T., Tedford C., Finlayson G.S. (2015). Effects of targeted delivery of propionate to the human colon on appetite regulation, body weight maintenance and adiposity in overweight adults. Gut.

[3] De Vadder F., Plessier F., Gautier-Stein A., Mithieux G. (2015). Vasoactive intestinal peptide is a local mediator in a gut-brain neural axis activating intestinal gluconeogenesis. Neurogastroenterol. Motil..

[4] Wong J.M.W., de Souza R., Kendall C.W.C., Emam A., Jenkins D.J. (2006). Colonic health: Fermentation and short chain fatty acids. J. Clin. Gastroenterol..

[5] Verbeke K.A., Boobis A.R., Chiodini A., Edwards C.A., Franck A., Kleerebezem M., Nauta A., Raes J., van Tol E.A., Tuohy K.M. (2015). Towards microbial fermentation metabolites as markers for health benefits of prebiotics. Nutr. Res. Rev..

[6] Wolever T.M.S., Spadafora P., Eshuis H. (1991). Interaction between colonic acetate and propionate in humans. Am. J. Clin. Nutr..

[7] Den Besten G., Lange K., Havinga R., van Dijk T.H., Gerding A., van Eunen K., Müller M., Groen A.K., Hooiveld G.J., Bakker B.M. (2013). Gut-derived short-chain fatty acids are vividly assimilated into host carbohydrates and lipids. Am. J. Physiol. Gastrointest. Liver Physiol..

[8] Frost G., Sleeth M.L., Sahuri-Arisoylu M., Lizarbe B., Cerdan S., Brody L., Anastasovska J., Ghourab S., Hankir M., Zhang S. (2014). The short-chain fatty acid acetate reduces appetite via a central homeostatic mechanism. Nat. Commun..

[9] Wright R.S., Anderson J.W., Bridges S.R. (1990). Propionate inhibits hepatocyte lipid synthesis. Exp. Biol. Med..

[10] Nishina P.M., Freedland R.A. (1990). Effects of propionate on lipid biosynthesis in isolated rat hepatocytes. J. Nutr..

[11] Roediger W.E. (1980). Role of anaerobic bacteria in the metabolic welfare of the colonic mucosa in man. Gut.

[12] Belcheva A., Irrazabal T., Robertson S.J., Streutker C., Maughan H., Rubino S., Moriyama E.H., Copeland J.K., Kumar S., Green B. (2014). Gut microbial metabolism drives transformation of msh2-deficient colon epithelial cells. Cell.

[13] Furusawa Y., Obata Y., Fukuda S., Endo T.A., Nakato G., Takahashi D., Nakanishi Y., Uetake C., Kato K., Kato T. (2013). Commensal microbe-derived butyrate induces the differentiation of colonic regulatory T cells. Nature.

[14] Arpaia N., Campbell C., Fan X., Dikiy S., van der Veeken J., deRoos P., Liu H., Cross J.R., Pfeffer K., Coffer P.J. (2013). Metabolites produced by commensal bacteria promote peripheral regulatory T-cell generation. Nature.

[15] Le Poul E., Loison C., Struyf S., Springael J.-Y., Lannoy V., Decobecq M.-E., Brezillon S., Dupriez V., Vassart G., Van Damme J. (2003). Functional characterization of human receptors for short chain fatty acids and their role in polymorphonuclear cell activation. J. Biol. Chem..

[16] Stoddart L.A., Smith N.J., Milligan G. (2008). International Union of Pharmacology. LXXI. Free Fatty Acid Receptors FFA1, -2, and -3: Pharmacology and Pathophysiological Functions. Pharmacology.

[17] Lin H.V., Frassetto A., Kowalik E.J., Nawrocki A.R., Lu M.M., Kosinski J.R., Hubert J.A., Szeto D., Yao X., Forrest G. (2012). Butyrate and propionate protect against diet-induced obesity and regulate gut hormones via free fatty acid receptor 3-independent mechanisms. PLoS ONE.

[18] Maslowski K.M., Vieira A.T., Ng A., Kranich J., Sierro F., Yu D., Schilter H.C., Rolph M.S., Mackay F., Artis D. (2009). Regulation of inflammatory responses by gut microbiota and chemoattractant receptor GPR43. Nature.

[19] Canfora E.E., Jocken J.W., Blaak E.E. (2015). Short-chain fatty acids in control of body weight and insulin sensitivity. Nat. Rev. Endocrinol..

[20] Macfarlane S., Macfarlane G.T. (2003). Regulation of short-chain fatty acid production. Proc. Nutr. Soc..

[21] Gietl E., Mengerink W., de Slegte J., Gibson G., Rastall R., van den Heuvel E. (2012). Factors involved in the in vitro fermentability of short carbohydrates in static faecal batch cultures. Int. J. Carbohydr. Chem..

[22] Salvador V., Cherbut C., Barry J.L., Bertrand D., Bonnet C., Delort-Laval J. (1993). Sugar composition of dietary fibre and short-chain fatty acid production during in vitro fermentation by human bacteria. Br. J. Nutr..

[23] Mortensen P.B., Holtug K., Rasmussen H.S. (1988). Short-chain fatty acid production from mono- and disaccharides in a fecal incubation system: Implications for colonic fermentation of dietary fiber in humans. J. Nutr..

[24] Laurentin A., Edwards C.A. (2004). Differential fermentation of glucose-based carbohydrates in vitro by human faecal bacteria—A study of pyrodextrinised starches from different sources. Eur. J. Nutr..

[25] Mortensen P.B., Nordgaard-Andersen I. (1993). The dependence of the in vitro fermentation of dietary fibre to short-chain fatty acids on the contents of soluble non-starch polysaccharides. Scand. J. Gastroenterol..

[26] Champ M.M.J. (2004). Physiological aspects of resistant starch and in vivo measurements. J. AOAC Int..

[27] Vogt J.A., Pencharz P.B., Wolever T.M.S. (2004). l-Rhamnose increases serum propionate in humans. Am. J. Clin. Nutr..

[28] Vogt J.A., Ishii-schrade K.B., Pencharz P.B., Wolever T.M.S. (2004). l-Rhamnose increases serum propionate after long-term supplementation, but lactulose does not raise serum acetate. Am. J. Clin. Nutr..

[29] Sanz M.L., Gibson G.R., Rastall R.A. (2005). Influence of disaccharide structure on prebiotic selectivity in vitro. J. Agric. Food Chem..

[30] Deville C., Gharbi M., Dandrifosse G., Peulen O. (2007). Study on the effects of laminarin, a polysaccharide from seaweed, on gut characteristics. J. Sci. Food Agric..

[31] Martens E.C., Lowe E.C., Chiang H., Pudlo N.A., Wu M., McNulty N.P., Abbott D.W., Henrissat B., Gilbert H.J., Bolam D.N. (2011). Recognition and degradation of plant cell wall polysaccharides by two human gut symbionts. PLoS Biol..

[32] Walker A.W., Duncan S.H., Leitch E.C.M., Child M.W., Flint H.J. (2005). pH and peptide supply can radically alter bacterial populations and short-chain fatty acid ratios within microbial communities from the human colon. Appl. Environ. Microbiol..

[33] Reichardt N., Duncan S.H., Young P., Belenguer A., McWilliam Leitch C., Scott K.P., Flint H.J., Louis P. (2014). Phylogenetic distribution of three pathways for propionate production within the human gut microbiota. ISME J..

[34] Kettle H., Louis P., Holtrop G., Duncan S.H., Flint H.J. (2015). Modelling the emergent dynamics and major metabolites of the human colonic microbiota. Environ. Microbiol..

[35] Adiotomre J., Eastwood M.A., Edwards C.A., Brydon W.G. (1990). Dietary fiber: In vitro methods that anticipate nutrition and metabolic activity in humans. Am. J. Clin. Nutr..

[36] Khan K.M., Edwards C.A. (2005). In vitro fermentation characteristics of a mixture of Raftilose and guar gum by human faecal bacteria. Eur. J. Nutr..

[37] Morrison D.J., Mackay W.G., Edwards C.A., Preston T., Dodson B., Weaver L.T. (2006). Butyrate production from oligofructose fermentation by the human faecal flora: What is the contribution of extracellular acetate and lactate?. Br. J. Nutr..

[38] Bourriaud C., Robins R.J., Martin L., Kozlowski F., Tenailleau E., Cherbut C., Michel C. (2005). Lactate is mainly fermented to butyrate by human intestinal microfloras but inter-individual variation is evident. J. Appl. Microbiol..

[39] Polyviou T., MacDougall K., Chambers E.S., Viardot A., Psichas A., Jawaid S., Harris H.C., Edwards C.A., Simpson L., Murphy K.G. (2016). Randomised clinical study: Inulin short-chain fatty acid esters for targeted delivery of short-chain fatty acids to the human colon. Aliment. Pharmacol. Ther..

[40] O’Keefe S.J.D., Li J.V., Lahti L., Ou J., Carbonero F., Mohammed K., Posma J.M., Kinross J., Wahl E., Ruder E. (2015). Fat, fibre and cancer risk in African Americans and rural Africans. Nat. Commun..

[41] Fässler C., Arrigoni E., Venema K., Brouns F., Amadò R. (2006). In vitro fermentability of differently digested resistant starch preparations. Mol. Nutr. Food Res..

[42] Kaur A., Rose D.J., Rumpagaporn P., Patterson J.A., Hamaker B.R. (2011). In vitro batch fecal fermentation comparison of gas and short-chain fatty acid production using “slowly fermentable” dietary fibers. J. Food Sci..

[43] Stewart M.L., Timm D.A., Slavin J.L. (2008). Fructooligosaccharides exhibit more rapid fermentation than long-chain inulin in an in vitro fermentation system. Nutr. Res..

[44] Valk V., van der Kaaij R.M., Dijkhuizen L. (2016). Characterization of the starch-acting MaAmyB enzyme from Microbacterium aurum B8.A representing the novel subfamily GH13_42 with an unusual, multi-domain organization. Sci. Rep..

